# Validation of the Barthel Index in Chinese nursing home residents: an item response theory analysis

**DOI:** 10.3389/fpsyg.2024.1352878

**Published:** 2024-04-30

**Authors:** Minyu Liang, Mei Yin, Bing Guo, Yichao Pan, Tong Zhong, Jieyi Wu, Zengjie Ye

**Affiliations:** ^1^School of Nursing, Guangzhou University of Chinese Medicine, Guangzhou, China; ^2^Assisted Living Facility, Home For The Aged Guangzhou, Guangzhou, China; ^3^Department of Vasculocardiology, Guangzhou First People's Hospital, Guangzhou, China; ^4^Department of Tumor Radiotherapy, Zhuhai People's Hospital (Zhuhai Hospital Affiliated with Jinan University), Zhuhai, China; ^5^School of Nursing, Guangzhou Medical University, Guangzhou, China

**Keywords:** Barthel Index, psychometric properties, nursing home residents, item response theory, differential item functioning

## Abstract

**Background:**

The Barthel Index (BI) is used to standardize the grading of assessments for clinical needs, insurance support, and long-term care resource allocation in China. However, its psychometric properties among nursing home residents remain unclear. Therefore, this study aims to assess and modify the psychometric properties of BI in nursing home residents.

**Methods:**

A total of 1,402 individuals undergoing evaluation in a nursing home facility in China were included in this study from November 2021 to November 2022. Correlations between items were examined to identify the potential multicollinearity concerns. The unidimensional item response theory (IRT) was used to validate and modify the single structure of BI. Furthermore, the logistic regression/IRT hybrid DIF detection method was conducted to assess differential item functioning (DIF) between the dementia group and the normal group.

**Results:**

The pairing of items 5 (“bowl control”) and 6 (“bladder control”) revealed a local dependence issue, leading to their consolidation. Items 56 (bowel and bladder control) and 9 (mobility) both displayed poor fit indices and underwent category collapsing. Through the application of the generalized partial credit model, the adjusted scale displayed better fit indices, demonstrating a robust discriminative power (DC >1.5) and orderly thresholds. Furthermore, non-uniform DIF was identified in item 2 (bathing) between the dementia group and the normal group.

**Conclusion:**

The modified BI demonstrated favorable psychometric properties and proved to be suitable for evaluating nursing home residents experiencing moderate functional impairment, which may provide a precise evaluation for long-term care resource allocation. Future studies could explore integrating supplementary measurements, such as objective indices, to assess a broader spectrum of functional statuses to potentially enhance the limited precision width observed in BI.

## Introduction

The Chinese population is on the brink of an aging era, and the percentage of individuals aged ≥65 years is projected to increase from 13% to 27.9% (180–380 million) between 2020 and 2050 (World Health Organization, 2018; China Development Research Foundation, 2020). The lifespan of individuals is correlated with low levels of physical activity and increased dependency. This eventually leads to a higher demand for long-term care needs, which was estimated at 45.30 million in 2020 and is expected to increase by 39.0% to 59.32 million by 2030 (World Health Organization, 2015; Zapata-Lamana et al., 2021; Gong et al., 2022; Musa et al., 2022; Parra-Rizo et al., 2022). However, family-based non-professional care is becoming less feasible due to the China's historical one-child policy, the prevalence of empty nesters amid urbanization, and the widespread migration of the population (Qian et al., 2018). Consequently, care provided by nursing home staff is emerging as an indispensable alternative. Long-term care resource allocation (i.e., conditions of access to nursing home and clinical care needs) is identified by standardized grading assessments (Zhang et al., 2018), which typically centers on the assessment of activities of daily life (ADLs) (Fortinsky et al., 1999; Hébert et al., 1999; Holanda et al., 2022; Jeppestøl et al., 2022). The original 10-item Barthel Index (BI) has gained recognition and been integrated into the China's standardized grading assessment system for evaluating general ADL function in older adults due to its communicability, simplicity, and ease of scoring (Dickinson, 1992; Bouwstra et al., 2019; Medical Administration and Medical Authority, 2020). Studies have highlighted favorable psychometric properties of BI when used for patients with stroke, Parkinson's disease, and older adults (Pashmdarfard and Azad, 2020). However, one study indicated that the reliability of BI fluctuated when it was used for evaluating severe disability (Sainsbury et al., 2005). Given that nursing home residents, typically above 70 years of age, often exhibit serious disability or dependence (Muszalik et al., 2021; Kashiwagi and Morioka, 2022; Zhao et al., 2022), questions arise regarding the suitability of BI for their evaluation. A previous study using BI-10 to assess 644 patients with dementia across 19 long-term care facilities in Thailand, Japan, and South Korea revealed compromised item fit, including item bias, redundancy, and narrow threshold widths, casting doubt on the suitability of BI for assessing dementia and indicating a need for modification (Yi et al., 2020). However, to date, no study has been conducted to validate the use of the 10-item BI for nursing home residents in mainland China. Therefore, it is imperative to promptly validate the psychometric properties of BI for assessing nursing home residents in mainland China.

The classical test theory (CTT) ha been widely used to assess the psychometric properties of scales, focusing on assessing construct validity and internal consistency. However, it overlooks measurement precision, leading to an incomplete understanding of scale's psychometric properties (Hunsley and Mash, 2008). In contrast, the item response theory (IRT) has emerged as a more comprehensive framework, offering deeper insights into internal consistency, factor structure, and measurement precision. This theory provides a precise psychometric framework that delineates interactions between individuals and items to determine if items effectively measure the intended population. Additionally, precision is typically demonstrated through item information curves or test information, highlighting the optimal discrimination location of an item or a scale across individual latent traits (Reckase, 2009). Moreover, IRT enables the detection of item- and test-level biases via differential item functioning (DIF) analysis, allowing for adjustments to mitigate or eliminate these biases (Teresi et al., 2012). Moreover, IRT assessed item functions, such as the discrimination ability and difficulty of each item, without relying on the sample, a capability that CTT lacks (Reckase, 2009; Teresi et al., 2012). Hence, compared to CTT, IRT offers more comprehensive information (e.g., item function, measurement precision) for instrument development and validation.

Therefore, the objective of this study is to validate the psychometric properties of the 10-item BI, tailoring their use to suit the specific context of nursing homes. The current study hypothesizes that the 10-item BI may have compromised psychometric properties for evaluating nursing home residents in mainland China and a modified version of the BI may serve as a suitable instrument.

## Methods

### Data and samples

The current study involved a secondary analysis of data obtained from an electronic evaluation system in a nursing home, encompassing 1568 residents enrolled consecutively from November 2021 to November 2022. The inclusion criteria were as follows: (1) individuals aged ≥60 years; (2) residents in a nursing home facility; (3) nursing home residents who had undergone evaluation. The exclusion criteria included participants with incomplete demographic or instrument data. As a result, 166 residents were excluded: 125 (8.4%) were not evaluated, and 41 (2.7%) had missing data. Finally, the study comprised 1,402 nursing home residents. Ethics approval was obtained from the ethics committee of the First Affiliated Hospital of Guangzhou University of Chinese Medicine (K-2023-046).

### Measures

#### Demographic evaluation

The demographic evaluation included several factors, such as sex, age, education, comorbidities, marital status, cognitive status, and the BI level grading.

#### Barthel Index

Mahoney and Barthel introduced the BI in 1965, comprising 10 items designed to assess activities feeding, bathing, grooming, dressing, using toilet, transferring (moving from the bed to the chair and back), mobility (on level surfaces), climbing stairs, and controlling bowel and bladder functions (Honey and Barthel, 1965). Among these, two items (items 2 and 3) feature dichotomous response categories, while six items (items 1, 4, 5, 6, 7, and 10) and two items (items 8 and 9) employ three- and four-graded response options, respectively. Total scores range from 0 to 100, with higher scores indicating greater levels of functional independence. In the current study, Cronbach's alpha was recorded at 0.95.

### Statistical analysis

First, a descriptive statistic method was employed to summarize the demographic characteristics of nursing home residents and to delineate the distribution patterns within item response categories. Second, the item correlation analysis and item residual correlation matrix were performed for potential item consolidation. Specifically, pairs of items exhibiting correlation coefficients (r1) > 0.95 indicated multicollinearity concerns (Tleyjeh et al., 2008). The residual correlation values exceeding 0.1 were defined as contravening the assumption of local independence (Reeve et al., 2007; Kline, 2011). Items showing signs of multicollinearity or failing to meet the criteria for local independence were considered for item consolidation. Third, IRT was performed to analyze the unidimensional structure of the scale based on previous research (Wang et al., 2020). A unidimensional generalized partial credit model (GPCM) was selected to analyze polytomous item responses. Its equation is as follows (Muraki, 1992):


$$\begin{array}{l} P\left(X_{\text{ikj}}=k|\theta_{\text{j}}\right)=\frac{\exp\overset{k}{\underset{h=0}{\sum }}\left[a_{i}\left(\theta_{j}-b_{ih}\right)\right]}{\overset{m_{i}}{\underset{c=0}{\sum }}\exp\overset{c}{\underset{h=0}{\sum }}\left[a_{i}\left(\theta -b_{ih}\right)\right]} \end{array}$$

X_*ikj*_ refers to the person (*j*)'s response in category *k* of item *i*. The probability (*P*) of X_*ikj*_ is defined as a logistic probability function and is determined by the slope or discrimination parameter vector (a_*i*_), item-category threshold parameter vector (b_*ih*_), and person latent trait parameter vector (θ_*j*_).

Adequate fit was defined by root mean square error of approximation (RMSEA) < 0.1, Tucker–Lewis index (TLI) >0.90, comparative fit index (CFI) >0.90, standardized root mean square residual (SRMSR) < 0.08, and low values of Akaike Information Criterion (AIC), Bayesian Information Criterion (BIC), and sample-adjusted BIC (SABIC) (Hu and Bentler, 1999; Byrne, 2006; Hooper et al., 2008). Additionally, Pearson's χ2 (S-X^2^) was used to identify misfit items and to determine the need for collapsing categories (Chalmers and Ng, 2017; Robinson et al., 2019).

The two crucial item parameters, namely discrimination and threshold/difficulty, were assessed based on the following criteria: discrimination was considered poor if it was < 0.5, moderate if it was between 0.5 and 1.0, good if it was within the range of 1.0–1.5, and excellent if it was >1.5. Threshold/difficulty was deemed good if it satisfied the requirement of monotonicity of item response (Reckase and McKinley, 1991). Subsequently, measurement accuracy was determined by the information function concerning BI. Finally, item- and test-level biases between the dementia group and the normal group were identified by DIFs using logistic regression/the IRT hybrid DIF method (Choi et al., 2011). The statistical process involved the use of R, Mplus, and SPSS.

## Results

### Demographic characteristics

Most residents (83.8%) did not have a diagnosis of dementia. Among them, 62.8% were female individuals, and the mean age was 81.32 years (standard deviation = 9.0). A vast majority (93.9%) of them had multiple comorbidities. Further demographic details are presented in Table 1.

**Table 1:** Demographic characteristics of nursing home residents (*n* = 1,402).

**Characteristics**		***N* (%)**
Sex	Female	881 (62.8)
	Male	521 (37.2)
Age (years)	< 80	544 (38.8)
	>=80	858 (61.2)
Education level	Middle school	916 (65.3)
	High school or technical secondary school	293 (20.9)
	College degree or above	193 (13.8)
Comorbidities	0	86 (6.1)
	1	708 (50.5)
	≥2	608 (43.4)
Cognitive status	Dementia disease	227 (16.2)
	No-dementia disease	1,175 (83.8)
Marital status	Having spouse	580 (41.4)
	No spouse	822 (58.6)
BI grading	0	197 (14.1)
	1	639 (45.6)
	2	130 (9.3)
	3	436 (31.0)

### Item distributions, correlations, and local independence

**Figure 1:**
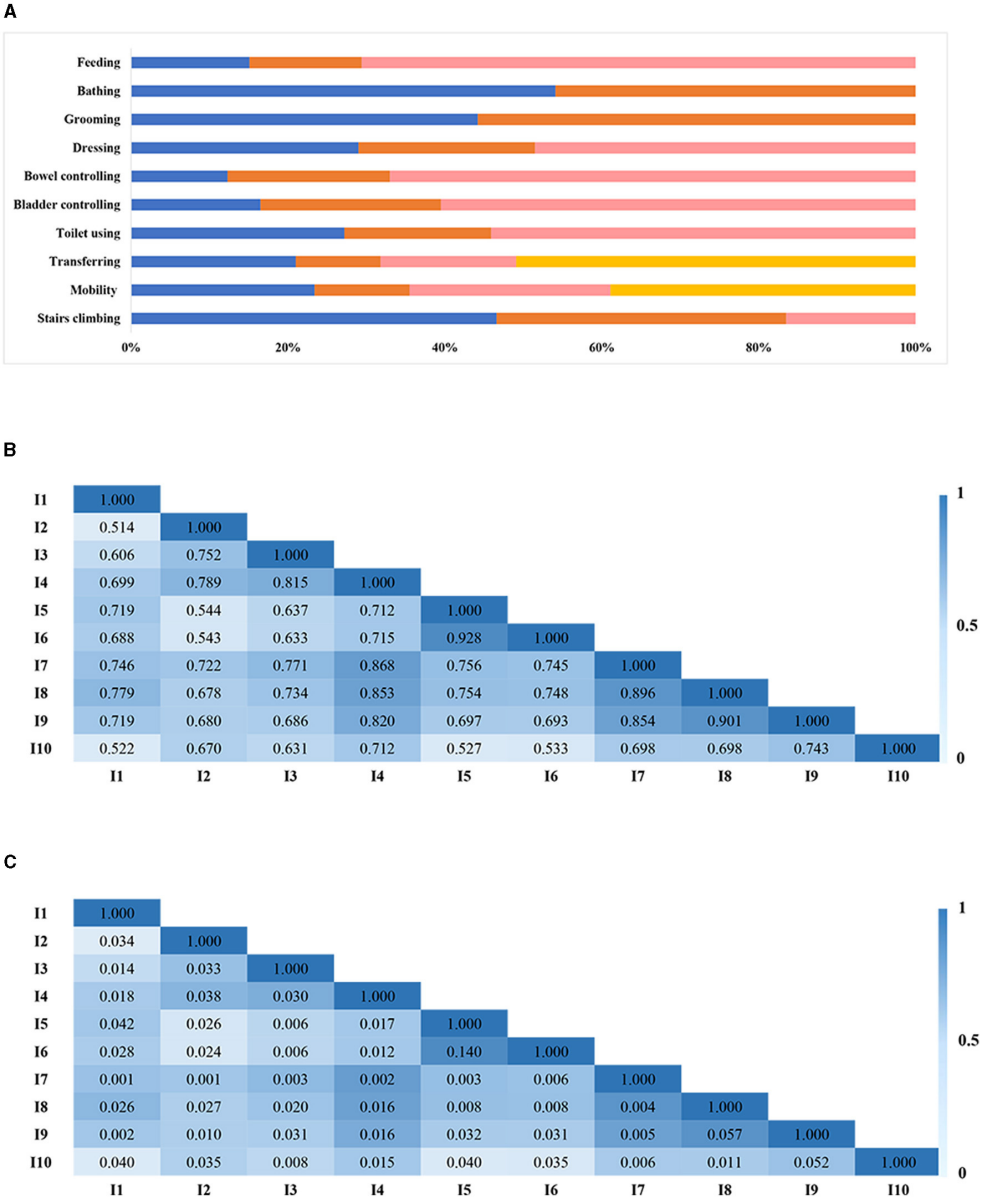
Item distribution **(A)**, item correlation **(B)**, and local independence **(C)**. **(B, C)** A deeper color suggested a stronger association. Its value < 0.10 indicated local independence satisfaction.

Figure 1A summarizes the item distributions. The correlation analysis revealed that the correlation coefficients (r1) among each item ranged from 0.514 to 0.928 (Figure 1B). No item exhibited multicollinearity. However, it is noteworthy that the residual correlation coefficient between item 5 (bowel control) and item 6 (bladder control) surpassed 0.1, indicating a violation of local independence (Figure 1C). Consequently, items 5 and 6 were combined into a new item (bowel and bladder control). The modified BI comprised nine items including feeding, bathing, grooming, dressing, bowel and bladder control, toilet usage, transferring, mobility, and stair climbing. The sample was randomly divided into two equal groups, namely the developmental sample (*N* = 701) and the validation sample (*N* = 701).

### Confirmation of the unidimensional structure

**Figure 2:**
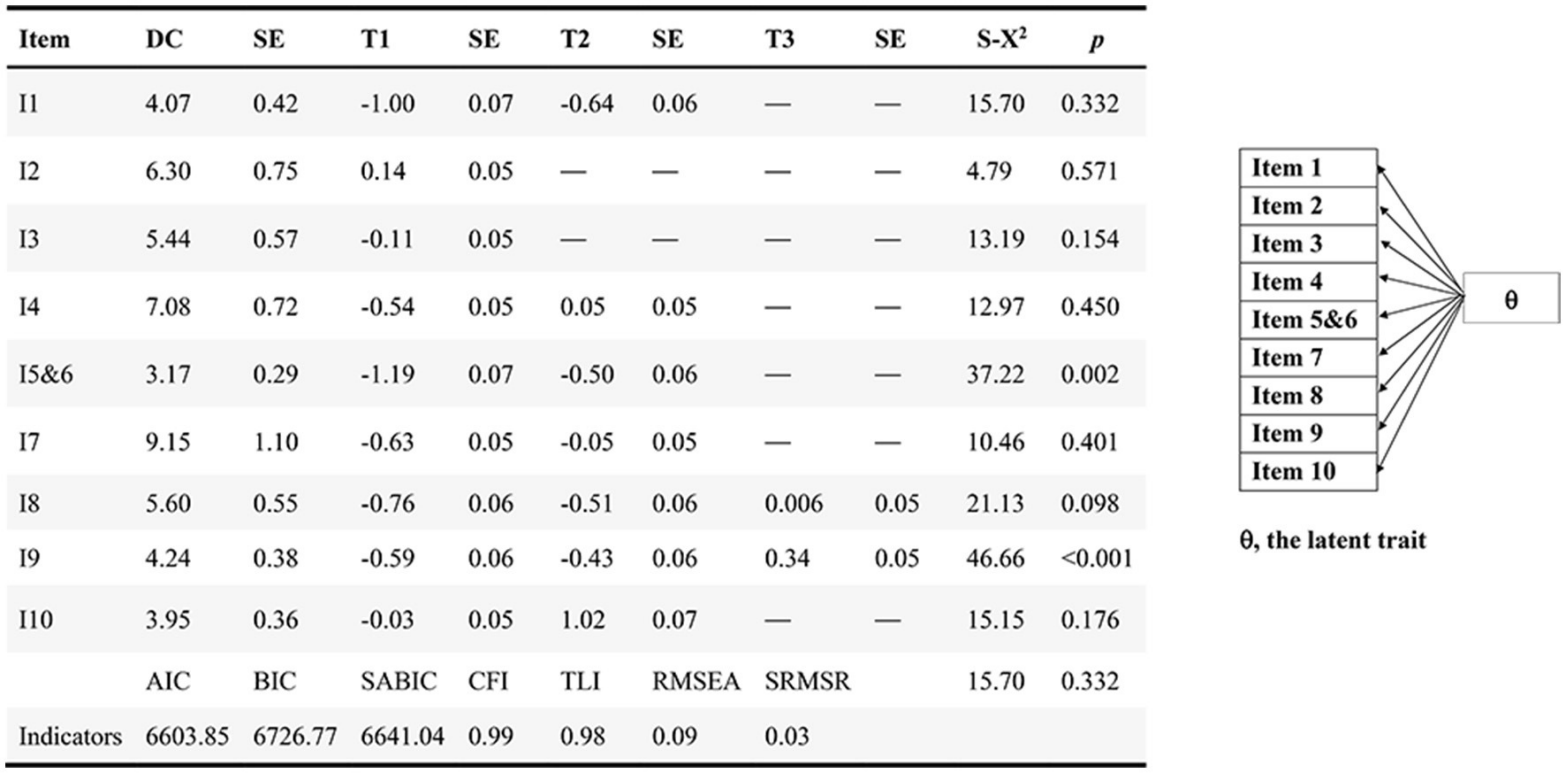
IRT analysis (development sample, *n* = 701).

In the developmental sample, the unidimensional structure GPCM met the good model fit criteria, with CFI > 0.95, TLI > 0.95, RMSEA < 0.1, and SRMSR < 0.08 (Figure 2). All items exhibited discrimination values >1.5, indicating robust discrimination among individuals across varying functional levels (Figure 2). Additionally, the thresholds displayed an orderly trend, escalating as the categories increased. Notably, item 56 (bowel and bladder control) and item 9 (mobility) were considered misfit (*p* < 0.05), suggesting the need for collapsing categories. Thus, the adjected categories with similar meanings were combined. For item 56, the adjacent rating categories of “occasional incontinence” and “complete incontinence” were integrated into a single category termed “incontinence”. Consequently, item 56 was restructured into two categories, namely “incontinence” and “voluntary”. As for item 9, the adjacent rating categories “wheelchair independent” and “walking with help” were combined into a single category named “some help from others or devices”. Thus, item 9 encompassed categories of “dependent”, “some help from others or devices”, and “independent”.

### Validation of the Barthel Index

**Figure 3:**
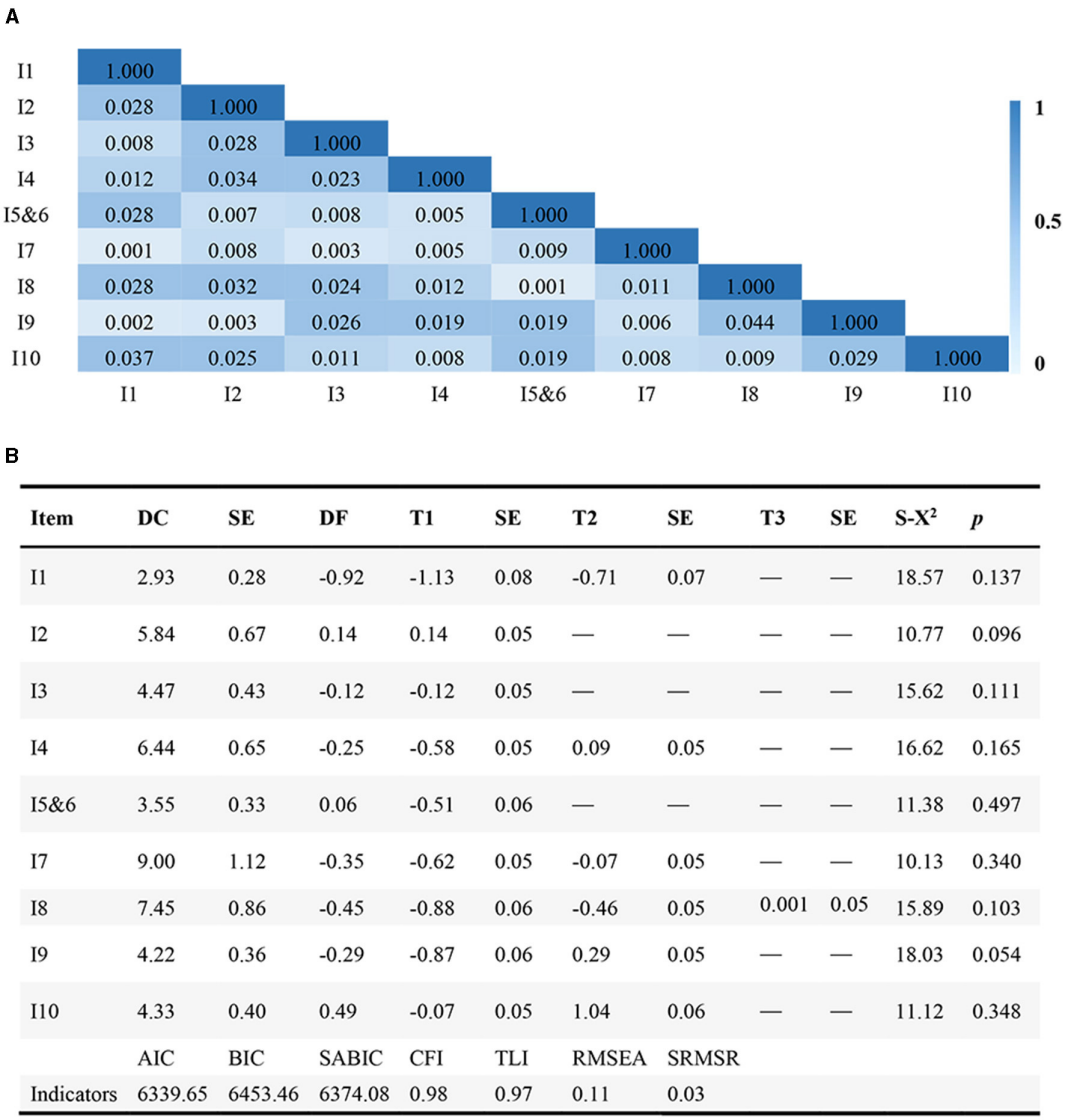
Local independence of the validation sample (*n* = 701) **(A)** and IRT analysis of the validation sample **(B)**. **(B)** A deeper color suggested a stronger item-pair residuals association. Its value < 0.10 indicated local independence satisfaction.

**Figure 4:**
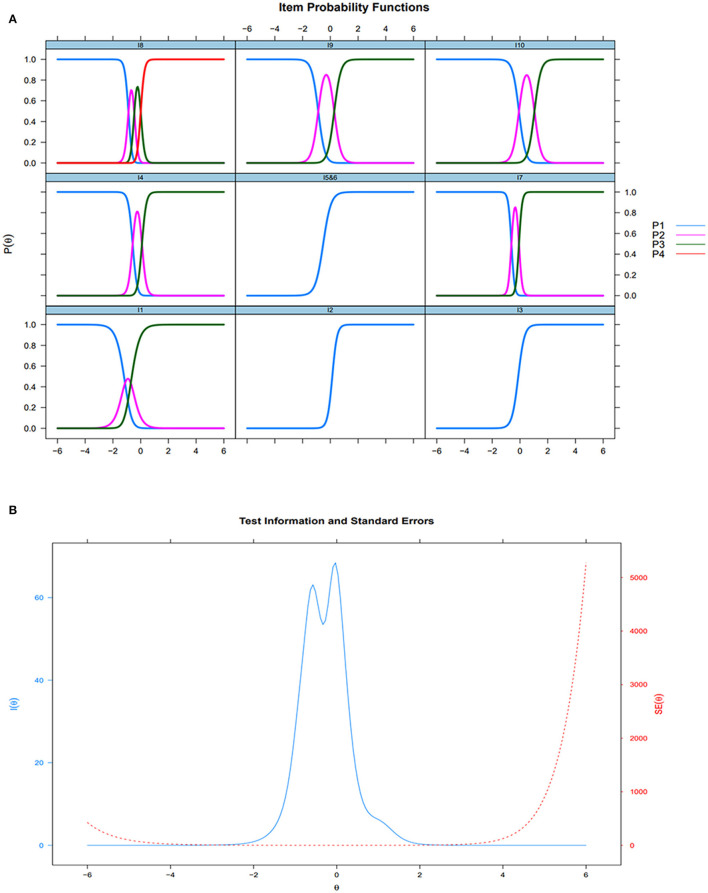
Items Characteristic Curves (validation sample, *n* = 701) **(A)** and Test Information and Test Standard Errors (validation sample, *n* = 701) **(B)**.

In the validation sample, the unidimensional structure GPCM was employed to validate the modified BI. The residual correlation coefficients for item pairs were below 0.10, indicating compliance with the local independence hypothesis (Figure 3A). While most model indices indicated a good fit (CFI=0.98, TLI=0.97, and SRMSR=0.03), the RMSEA index exceeded 0.1. Additionally, the model displayed a robust discriminative power (DC >1.5), order thresholds, and no instances of item misfit (Figure 3B). Threshold widths for the items ranged from 0.42 to 1.16 logits. Item 10 (stair climbing) emerged as most challenging, followed by item 2 (bathing), while item 1 (feeding) was the easiest, followed by item 8 (transferring). Items 3 (grooming), 4 (dressing), 56 (bowel and bladder control), 7 (toilet usage), and 9 (mobility) demonstrated closely located difficulties (DF = −0.35–0.06). Illustrations of item characteristic curves (ICCs), test information, and test standard errors are presented in Figure 4. The item information covered latent trait (θj) levels from −3 to 3, with the majority concentrated between −1 and 1. This indicates that the modified BI offered optimal measurement accuracy for respondents with moderate functional impairment, rather than those at minimum or maximum function trait levels.

### Differential item functioning

**Figure 5:**
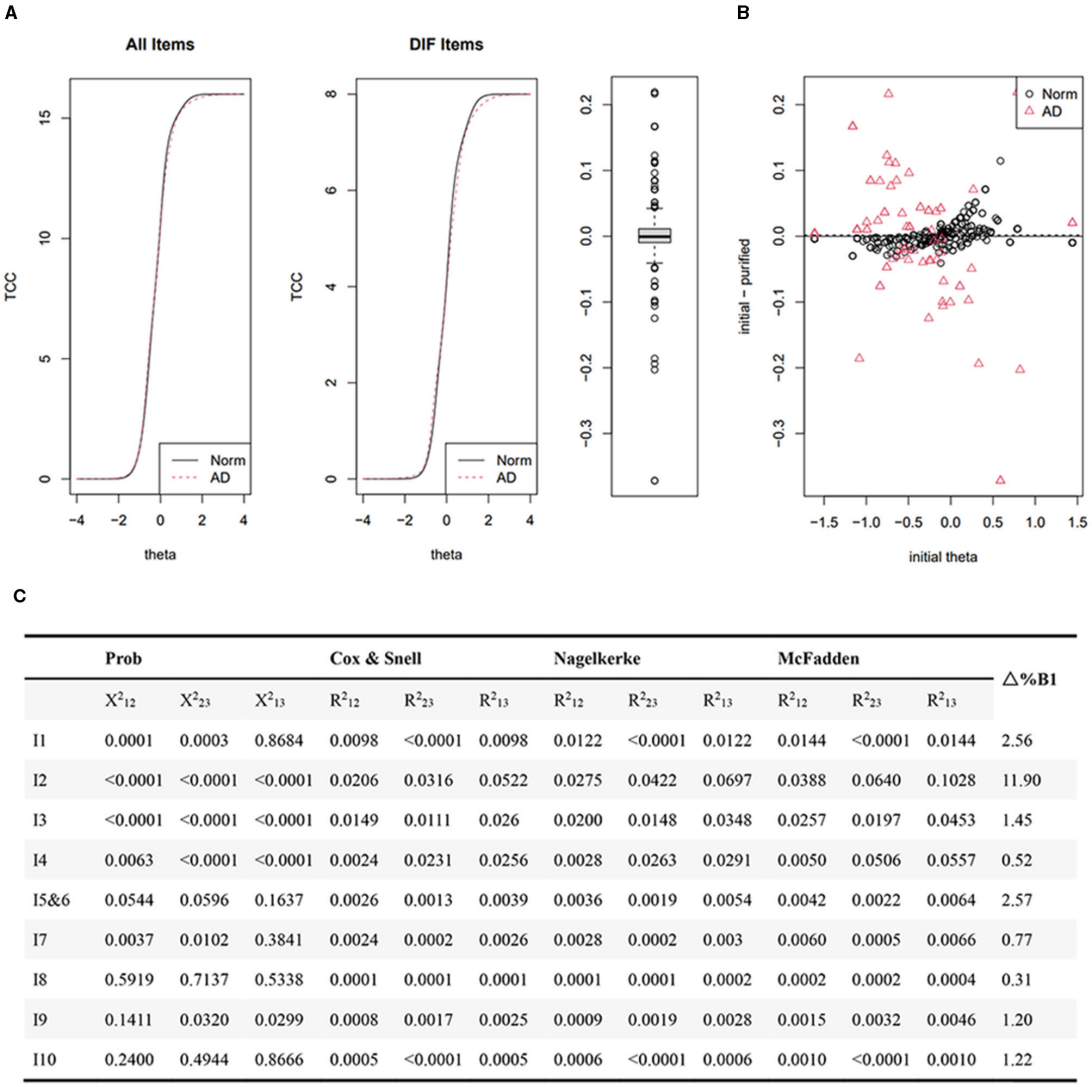
Differential item functioning analysis employing iterative hybrid ordinal logistic regression/item response theory and Monte Carlo simulations. *P*-values of Chi-square detect uniform and non-uniform DIF on the basis of comparing Models 1 and 2, and Models 2 and 3 respectively. *P* values < (0.01/9 = 0.0011) indicated statistically significant. R^2^ difference (Cox and Snell, Nagelkerke and McFadden) for detecting uniform and non-uniform DIF comes from difference between Models 1 and 2, and 2 and 3 respectively. Δ%B1=|B1(Model 1) – B1(Model 2)/B1(Model 1)|^*^100%. R^2^ < 0.02 is considered as ignored based on Cohen's guideline; Δ%B1 is considered as ignored. Test characteristic curves of all items and items with differential item functions **(A)**; The difference of individual functional levels between the dementia and normal groups **(B)**; Monte Carlo simulation-based thresholds **(C)**.

The dementia group and the normal group revealed a minimal distinction in the total expected score as depicted by the test characteristic curves (TCCs) of all items (both items with and without DIF) and DIF items (Figure 5A). Individual functional levels displayed no statistically significant difference between the dementia and normal groups (Figure 5B). Monte Carlo simulation-based thresholds (Figure 5C) identified non-uniform DIF in items 1 (feeding), 2 (bathing), 3 (grooming), and 4 (dressing) based on a chi-squared value (*p* < 0.0011). However, the differences in items 1, 3, and 4 were deemed negligible, given the proportionate β1 change (< 5%) and R2 change (< 0.02). Only item 2 (bathing) exhibited non-uniform DIF (*p* < 0.0001, Δ%B1 = 11.90%, R2 change > 0.02).

## Discussion

The present study employed the IRT to validate the psychometric properties of the BI for nursing home residents, aiming to gather more detailed item information compared to the CTT (Reckase, 2009). This study represents the first large-sample examination of BI's psychometric properties among nursing home residents in mainland China. The findings confirmed our hypothesis that the 10-item BI showed unsatisfactory psychometric properties (violation of local independence and item misfit). While the modified BI demonstrated optimal psychometric properties: the unidimensional structure indicated a good model fit, the items possessed robust discriminative power, the thresholds displayed an orderly trend, and no item was misfit. Thus, the modified BI may present as the better instrument for evaluating nursing home residents. It retained nine items but still assessed ten aspects of ADLs, as some items and categories were consolidated, instead of being discarded, to achieve the optimal psychometric properties and preserve the maximum information from the original scale. Moreover, the modified BI showed high measurement accuracy for residents with moderate functional impairment rather than those with minimum or maximum function levels.

While the fit indices in the validation samples displayed inconsistencies regarding model fitting (CFI and TLI >0.95 indicated a good model fit, while RMSEA >0.1 suggested a poor fit), the one-factor GPCM remained the optimal model. A study conducted by Keke et al. suggested that strong correlations among observed variables might yield a “good” CFI but a “bad” RMSEA, emphasizing the importance of balance among various fit indices (Lai and Green, 2016). In this study, item pairs exhibited strong correlations (r1 = 0.51–0.93), contributing to the unfavorable RMSEA performance. Although making potential modifications to the model, such as excluding highly correlated item pairs, might yield a “better” RMSEA, it would result in a loss of valuable item information. Therefore, despite the compromised RMSEA due to substantial item correlations, the one-factor GPCM remained the optimal model in this study.

Ensuring adherence to the local independence hypothesis is essential for accurate parameter calculation. In this study, item pairs (items 5 and 6) violated the local independence hypothesis (>0.1) and were combined into a new item (bowel and bladder control). This finding aligns with previous research indicating that the mean thresholds for “controlling bladder” and “controlling bowel” were nearly identical, suggesting potential redundancy between these items (Yi et al., 2020). Furthermore, physiological mechanisms suggest that bladder and bowel contraction are controlled by the pelvic musculature, potentially leading to concurrent urinary and bowel dysfunction (Hubscher et al., 2021; Kajbafzadeh et al., 2021). Among nursing home residents, who are typically aged over 81 years and have decreased contraction ability of the pelvic musculature, there exists a likelihood of concomitant urinary and bowel dysfunction. Hence, consolidating “bladder control” and “bowel control” into a single item is warranted.

Regarding item fit, items 5, 6, and 9 displayed misfit. This misfit can stem from inappropriate item definitions, poor category definitions, or an excessive number of choices, leading to discordant participant responses (Andrich and Luo, 2003). Common strategies to address this issue involve eliminating misfit items or collapsing categories. Collapsing categories is the primary option to preserve more information. Following the collapsing of categories, item 56 comprises two categories “incontinence” and “voluntary”. This action was corroborated by prior research using the BI for assessing long-term care residents, which indicated the limited utility of the second category (“occasional incontinence”) within items related to bowel and bladder control for discriminating latent traits among individuals (Liu et al., 2015). In nursing home settings, residents experiencing complete incontinence typically use incontinence products and are assessed under the “complete incontinence” category. Conversely, those with intact sphincter control manage themselves and are assessed as having “voluntary” bowel and bladder control. Occasional incontinence poses a challenge as it often goes unnoticed until caregivers discover wet bed sheets (Sayabalian et al., 2019). Such instances might be classified as complete incontinence due to heavy caregiving burdens and workforce shortages in nursing homes, or they might go undetected, leading to categorization as voluntary bowel and bladder control. Consequently, the category of occasional incontinence becomes ambiguous. Thus, “incontinence” (consolidating categories of occasional and complete incontinence) and “voluntary” emerge as more suitable options for item 56 (bowel and bladder control) in nursing home assessments. Similarly, item 9 (mobility) exhibited misfit. The adjacent categories of “wheelchair independent” and “walking with help” might be ambiguous as many nursing home residents prefer wheelchairs despite being capable of walking short distances. Collapsing these two categories into a new category (“some help from others or device”) rendered item 9 compatible with the GPCM. This aligns with previous studies employing the Rasch analysis, which also suggested the potential ambiguity of the “mobility” item's second (wheelchair independent) and third (walking with help) categories leading to model misfit (van Hartingsveld et al., 2006; Yi et al., 2020). Therefore, collapsing categories aligns with the GPCM analysis and practicality within nursing home settings.

In terms of item functions, all nine items exhibited excellent discrimination, implying their robust ability to distinguish between varying functional levels. No disorderly thresholds were observed, indicating the suitability of the categories within the BI. Moreover, the maximum information was concentrated within the latent trait (θj) ranging from −1 to 1, while minimal information function was detected at latent trait (θj) values near −3 or 3. This indicates that the BI possesses a narrow assessment precision width, effectively discriminating among nursing home residents with moderate functional impairments but lacking the capacity to differentiate residents with minimal functional limitations or severe functional impairments. Similarly, the 15-item BI also demonstrated a narrow assessment precise width (−1 < θ < 1) (Zhao et al., 2022). Furthermore, reports of ceiling effects (>15% of the sample attaining the maximum score) and floor effects (>15% of the sample scoring the minimum) have been reported across different populations, such as patients with stroke (Balu, 2009), patients in the intensive care unit (Reis et al., 2021), and older hospitalized patients with cognitive spectrum disorders (Braun et al., 2021). These observations suggest an inherent limitation in the BI's measurement due to its focus on a limited range of ADLs and the absence of assessment beyond basic ADLs (Bouwstra et al., 2019). Consequently, this restricted range of individual locations within the BI might lead to a narrow precision width. The findings of the current study align with the observation that the BI comprises ADL items encompassing individual locations ranging from−1 to 1, offering precise measurement specifically for individuals experiencing moderate functional impairment. In our study, items such as “grooming”, “dressing”, “bowel and bladder control”, “toilet usage”, and “mobility” exhibited closely aligned difficulty levels, indicating potential redundancy among these items, prompting consideration for their removal. However, when a 5-item BI was developed from the original 10-item BI by eliminating redundant items, a significant loss of item information occurred, leading to unsatisfactory recommended reliability criteria (Hobart and Thompson, 2001). Hence, retaining these items with similar difficulty levels is essential to maintain the integrity of the scale structure and to preserve item information.

Regarding measurement bias, prior reports have not addressed DIF between the dementia and normal groups concerning the BI. DIF highlights a potential bias in measurement for each item. In this study, only non-uniform DIF was identified in item 2 (bathing). This suggests a potential disparity in bathing abilities between the dementia and normal groups, a trend supported by previous studies indicating a higher dependency on bathing among individuals with dementia (Liu et al., 2015). Despite this, minimal differences surfaced between the dementia and normal groups in the total expected score across all items, including those with DIF. This implies that any distortion in the scale due to the DIF item is likely insignificant.

### Implications for research and clinical practice

First, examining item difficulty through the GPCM could elucidate the sequence of ADL decline among nursing home residents, furnishing valuable insights into potential functional impairments. For instance, item 10 (stair climbing) emerged as most challenging, followed by item 2 (bathing), while item 1 (feeding) posed the least difficulty, followed by item 8 (transferring). This pattern suggests that stair climbing and bathing constitute the initial loss of ADLs while feeding and transferring represent the final stages of decline in ADLs. These findings are consistent with those of previous studies indicating that older individuals find “climbing stairs” and “bathing” most challenging due to higher oxygen consumption (Bauer et al., 2022), whereas “feeding” and “transferring” rank among the easiest ADLs (Liu et al., 2015). The inability to perform the simplest ADLs (such as feeding and transferring) indicates severe functional impairment, while difficulty with the most challenging tasks (such as stair climbing or bathing) suggests milder functional impairment, offering insights for tailored individual care based on the function level. Second, despite the widespread clinical use of the BI, there remains a lack of comprehensive information on its psychometric properties for nursing home residents. This study, benefitting from a large sample size, effectively used IRT, resulting in more precise and reliable outcomes. Using the GPCM analysis and drawing from nursing home practices, modifications were made to the BI, particularly combining items 5 and 6, and collapsing categories in items 56 and 9 to modify BI. These adaptations aim to align the BI with the nursing home environment while preserving maximum information. Moreover, the diverse pool of eligible participants in nursing homes, spanning a wide spectrum of functional statuses without specific diagnostic restrictions or limited comorbidities, ensures that the study outcomes are broadly applicable across populations. Third, while the BI provides precise measurements for individuals with moderate functional impairment, it also provides limited information for individuals with mild or severe functional impairments. This finding bears significance for long-term care insurance policies, which often rely on BI assessments. Supplementing the BI with additional measurements, such as objective indices targeting mild or severe functional impairments, should be integrated into the long-term care assessment system. Finally, while the non-uniformity of item 2 did not significantly affect the overall scale performance, it highlights the necessity of further exploring minimal functional differences between the dementia and normal groups. A previous study demonstrated that employing a semi-structured interview focusing on ADL contents could facilitate the recognition of minimal impairments, enhancing precision in assessing daily functioning, including coordinated actions, proper execution, and completion levels (Cornelis et al., 2017). Crafting evaluation protocols tailored for assessing dementia-related groups could enhance the detection of minimal functional changes.

### Limitations

Certain limitations warrant consideration in this study. First, although the nurse administering the BI underwent prior training, the specific characteristics of these nurses were neither recorded nor accounted for, potentially introducing rater bias. Second, the narrow precision width of the BI is another limitation, resulting in less precise evaluations at the minimum and maximum levels of ADLs. Future research endeavors could integrate the BI with objective assessment tools to complement these limitations. Third, the sample size of individuals with dementia comprised < 20% of the total sample. Further studies with larger dementia cohorts are necessary to validate DIF between the dementia and normal groups. Finally, among the 1,402 residents included, 166 were excluded for various reasons. This exclusion could potentially introduce selection bias due to unknown differences in their functional levels.

## Conclusion

The modified BI demonstrated favorable psychometric properties and proved to be suitable for evaluating nursing home residents experiencing moderate functional impairment, which may provide precise evaluation for long-term care resource allocation. Future studies could explore integrating supplementary measurements, such as objective indices, to assess a broader spectrum of functional statuses to potentially enhance the limited precision width observed in the BI.

## Data availability statement

The original contributions presented in the study are included in the article/supplementary material, further inquiries can be directed to the corresponding author/s.

## Ethics statement

The studies involving humans were approved by the First Affiliated Hospital of Guangzhou University of Chinese Medicine Ethics Committee. The studies were conducted in accordance with the local legislation and institutional requirements. The participants provided their written informed consent to participate in this study.

## Author contributions

ML: Conceptualization, Data curation, Formal analysis, Investigation, Methodology, Project administration, Software, Writing – original draft. MY: Conceptualization, Data curation, Investigation, Project administration, Resources, Writing – review & editing. BG: Conceptualization, Data curation, Investigation, Project administration, Resources, Writing – review & editing. YP: Conceptualization, Data curation, Formal analysis, Methodology, Writing – review & editing. TZ: Conceptualization, Data curation, Writing – review & editing. JW: Conceptualization, Data curation, Writing – review & editing. ZY: Conceptualization, Supervision, Validation, Writing – review & editing.
